# Genetic typing and intrafamilial transmission of human T-lymphotropic virus type 1 in non-endemic areas of China

**DOI:** 10.3389/fmicb.2023.1288990

**Published:** 2023-10-18

**Authors:** Huimin Ji, Le Chang, Ying Yan, Huizhen Sun, Yi Liu, Lunan Wang

**Affiliations:** ^1^National Center for Clinical Laboratories, Beijing Hospital, National Center of Gerontology, Institute of Geriatric Medicine, Chinese Academy of Medical Sciences, Beijing, China; ^2^Beijing Engineering Research Center of Laboratory Medicine, Beijing Hospital, Beijing, China; ^3^63750 Military Hospital of the People's Liberation Army, Xi'an, Shaanxi, China; ^4^Graduate School, Peking Union Medical College, Chinese Academy of Medical Sciences, Beijing, China

**Keywords:** T-cell lymphotropic virus type 1/2 (HTLV-1/2) HTLV-1/2, blood donors, phylogenetic analysis, intrafamilial transmission, sexual transmission

## Abstract

The origin and intrafamilial transmission of Human T-Lymphotropic Virus Type 1 (HTLV-1) in non-endemic populations such as China is still unknown. In this study, donors from blood banks/centers in China (including 28 provinces and Shenzhen city) during 2019 and 2021 were screened for HTLV-1/2 antibody, and all the reactive samples were tested using a line immunoassay (LIA) and quantitative polymerase chain reaction (qPCR). Samples that can be detected using qPCR were amplified and sequenced for the long terminal repeat (LTR) region. The positive donors were contacted to identify their relatives. As a result, 4,451,883 blood donors were totally tested, and 50 of them were confirmed to be HTLV-1/2 positive. Viral LTR sequences genotyped from 26 HTLV-1 carriers demonstrated that all had the HTLV-1a genotype, of which Transcontinental and Japanese subgroups accounted for half each. There were 17 family members of 11 index donors detected, and the HTLV-1 infection rate in the spouses of male index donors (83.3%, 5/6) was significantly higher than that in the husbands of female index donors (0.0%, 0/4). However, 7 children of HTLV-1 positive women were tested and found negative. Therefore, our findings indicated that HTLV-1 is spreading silently from high-endemic to low-endemic areas in China. To prevent further HTLV-1/2 transmission, an efficient HTLV-1/2 screening strategy and counseling of the virus carriers are essential.

## Introduction

Human T-cell lymphotropic virus type 1 (HTLV-1) is the etiological agent of two severe diseases: adult T-cell leukemia (ATL) and HTLV-1-associated myelopathy/tropical spastic paraparesis (HAM/TSP) (Poiesz et al., 1980; Yoshida et al., 1984; Gessain et al., 1985). It is also thought to cause local and systemic inflammatory diseases, including infective dermatitis, uveitis, myopathies, arthritis, and bronchiectasis, but data on its prevalence are limited (Schierhout et al., 2020). Three other HTLV-related viruses (types 2 to 4) have been detected, but only HTLV-2 has been linked to rare human diseases (Nicolás et al., 2015). Most of the HTLV-1/2 infected subjects are rarely aware of infection, even though some mild symptoms occur, becoming viral reservoirs and spreaders. At least 5–10 million people are reported to be infected with HTLV-1 worldwide, but this estimation might be underestimated because prevalence data are mostly based on studies of blood donors, pregnant women, sex workers, or injecting drug users (Gessain and Cassar, 2012). The areas with the highest prevalence are southwestern Japan, the Caribbean islands, Central Africa, and South America (Ishak et al., 2020). In China, it is a non-endemic area, with a similar prevalence in Korea (7/100,000) and Hong Kong China (4.1/100,000) (Kwon et al., 2008; Chan et al., 2017). But for Fujian Province, it has a relatively high prevalence of 36.24/100,000 compared with other areas of China; however, it was still much lower than those in Japan (ranging from 0.5% to 5.8%), the Caribbean area (ranging from 1.7 to 17.4%), or western Africa countries (ranging from 1 to 2%), but similar to that of Taiwan (5.8/10,000) (Nicolás et al., 2015; Xie et al., 2015; Chang et al., 2021). The WHO has recommended that screening for HTLV-1/2 in blood donors should be performed according to local epidemiological evidence. Therefore, 100% of blood donors in areas with relatively high prevalence in other regions in China (higher than 3//100,000) and 10~30% of blood donors in areas with moderate prevalence (higher than 1/100,000 and lower than 3/100,000) are screened for HTLV-1/2 antibody in China from 2022, which will lighten the workload, reduce the screening cost, and decrease the transmission risk by blood transfusion as much as possible.

HTLV-1 has been classified into seven major subtypes (1a−1g), predominantly based on nucleotide sequence analysis of its long terminal repeat (LTR) region. The HTLV-1a subtype is distributed worldwide and can be further divided into five subgroups based on the geographical localization of HTLV-1 infection: A-Transcontinental, B-Japanese, C-West African, D-North African, E-Black Peruvian, and F-Senegalese (Afonso et al., 2019). The low variability and clustering of HTLV-1 in certain geographic areas make molecular analysis an avenue for investigating the origin and dissemination of HTLV-1. However, in China, only the HTLV-1 isolates from Fujian and Guangdong provinces were sequenced and analyzed, indicating the presence of HTLV-1a.A was the predominant subgroup (Xie et al., 2015; Liao et al., 2021). However, based on our previous data, HTLV-1-infected cases in China were not only restricted to the southeastern areas; consequently, the subtypes and origins in non-endemic regions remain unclear (Chang et al., 2021).

HTLV-1 is mainly transmitted by mother-to-child and sexual contact as well as transmitted by the transfusion of non-leukocyte-depleted blood components, liver, kidney, and lung transplantation (Verdonck et al., 2007). In developed countries, the use of contaminated needles by drug users appears to be the main risk of infection, but in developing countries, intrafamilial transmission is the main route of transmission. High intrafamilial transmission rates were described in the endemic areas of Japan (Iwanaga, 2020), Taiwan (Lu et al., 2001), Brazil (Ishak et al., 2020), and Argentina (Berini et al., 2007), where they ranged from 38 to 67.9%. Children breastfed for more than 6 months have an increased risk of infection, and women are more likely to be infected by unprotected sexual intercourse due to the high concentration of infected lymphocytes in semen (da Costa et al., 2013). However, there are few published data on the family transmission of HTLV-1 in China, and the intrafamilial transmission rate is difficult to estimate.

Therefore, in this study, we screened and confirmed HTLV-1/2 infections among blood donors from non-endemic regions of China to further understand the actual infection situation and to characterize the epidemiologic patterns of HTLV-1 infections. Additionally, we tested the donor's family members for HTLV-1 infection to investigate the intrafamilial transmission of HTLV-1 in this population.

## Materials and methods

### Samples and study design

**Figure 1:**
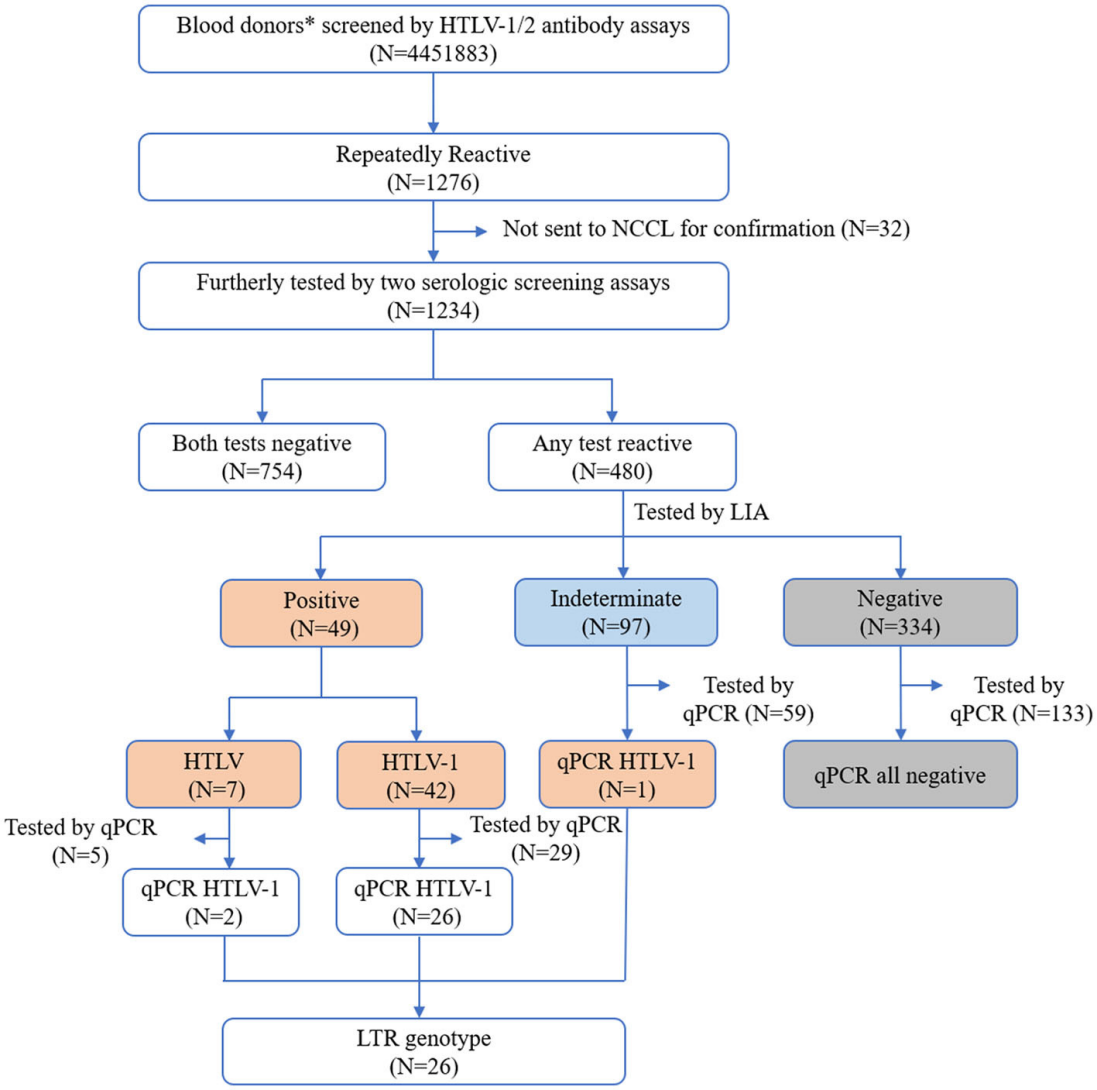
Screening and confirmation algorithm of HTLV infection. *Also include 12,083 healthy individuals from the Shandong Blood Hematopoietic Stem Cell Bank. NCCL, National Center for Clinical Laboratories.

All the specimens were collected from eligible donors from blood banks/centers in 28 provinces and Shenzhen city in China from January 2019 to December 2021, and 12,083 samples from the Shandong blood hematopoietic stem Cell Bank were also included. The initially reactive samples were delivered at 2–8°C or frozen conditions to the National Center for Clinical Laboratories (NCCL) for confirmation. According to the confirmatory process (Figure 1), all the initially reactive samples were simultaneously tested by two screening assays in the NCCL. Samples reactive to any one of the two tests were further tested by a line immunoassay (LIA) (INNO-LIA HTLV I/II score, Fujirebio, Japan).

Samples with limited plasma volume that were not sufficient for all assays' tests were excluded from our study. Enrolled samples with whole blood available were further quantified by qPCR established in our laboratory. Samples that can be detected using qPCR were also amplified for the LTR region of HTLV-1 by nested PCR. In addition, blood donors with positive results were recommended to collect blood samples from their relatives and test them using the same detection process. The positive donors were also asked to fill out a questionnaire about their personal experiences of probably being infected with HTLV-1/2 and some of the HTLV-1-related syndromes they have suffered.

### Immunological tests

Samples were initially screened for HTLV1/2 antibodies using one of the following three commercially available ELISA assays, including Wantai HTLV-1/2 antibody ELISA kits (Wantai BioPharm, China), Murex HTLV I+II (Diasorin S.p.A., United Kingdom), and Foresight HTLV-1/2 antibody ELISA kits (Acon Biotech, China). Reactive specimens underwent retests by the same assay, and any one of the two-round retests that were reactive would be defined as an initially anti-HTLV-1/2 reactive specimen and was sent to the NCCL for further confirmation.

Two different serologic assays, namely, Elecsys HTLV-I/II (Roche Diagnostics, Germany) and Murex HTLV I+II (Diasorin S.p.A., UK), were performed on one sample simultaneously in NCCL. Any sample that reacted to one of the two assays was confirmed and discriminated by the line immunoassay (INNO-LIA HTLV I/II Score, Fujirebio, Japan).

### Quantitative PCR and nested PCR

DNA was extracted from the whole blood of each blood sample by the Tiangen Magnetic Blood Genomic DNA Kit (Tiangen Biotech, China). HTLV-1 proviral DNA was detected and quantified as previously described (Ji et al., 2023). A pair of primer sets and a TaqMan probe were used to target the HTLV-1 *pol* region. The RPPH1 gene as an internal control was also amplified simultaneously. The primers and probes we used are listed in Supplementary Table S1. Standard curves were generated from recombinant plasmid DNA with HTLV-1 pol and RPPH1 sequences. The normalized HTLV-1 proviral load (PVL) was calculated as the ratio of (HTLV-1 DNA average copy number/RPPH1 average copy number) × 2 × 100 leukocytes, expressed as the number of HTLV-1 copies per 100 PBMCs. The detection limit for the HTLV-1 pol region was estimated to be 2.5 copies per 10,000 host leukocytes with a 95% hit rate.

The LTR region of HTLV-1 was amplified using a nested PCR as previously described (He et al., 2001). The obtained PCR products were purified using the Tiangen PCR purification kit (Tiangen Biotech, China) according to the manufacturer's instructions. Then, purified amplicons were directly sequenced with Sanger sequencing.

### HTLV-1 phylogenetic analysis and subtyping

All of the sequences of LTR regions were edited with the Geneious 9.1.4 software. These sequences were then aligned with 32 HTLV-1 sequences obtained from the Genbank in MEGA 7.0.26 (Mota et al., 2007; Afonso et al., 2019; Pashabayg et al., 2020). The nucleotide alignments were used to construct a phylogenetic tree for HTLV-1 classification using the maximum likelihood approach (bootstrap resampling test with 1,000 replicates) in the MEGA version 7.0.26 software. Phylogenetic trees were further optimized using the FigTree 1.4.3 software.

### Ethics approval

This study was approved by the institutional review boards of every participating blood bank. Written informed consent is routinely obtained at the time of donation from all of the donors. As for the positive donors, additional written informed consent, which was approved by the Beijing Hospital Ethics Committee (approval notice number 2017BJYYEC-138-01), was signed by them. If they agreed to allow their relatives to be tested, then the family members would be contacted and they would need to sign written informed consent.

### Statistical analysis

The difference between proportions was evaluated using the χ^2^ test. *P* < 0.05 was considered statistically significant. SPSS software version 17.0 was used for the statistical analyses.

## Results

### Confirmatory results

A total of 4,451,883 blood samples were initially screened for anti-HTLV-1/2 antibodies, and 1,276 were repeatedly reactive. Except for 32 samples that were not sent to NCCL, the other 1,234 were further confirmed according to the process described above. A total of 754 samples were not reactive in two additional screening assays (Elecsys HTLV-I/II or Murex HTLV I+II), and 480 were reactive in at least one screening assay. After confirmation by LIA, 42 samples were finally identified as HTLV-1 positive, 7 were HTLV positive, 97 were indeterminate, and 334 were negative. Most LIA-indeterminate samples expressed a single gp21 I/II pattern (95.9%, 93/97), while the others displayed p19 I/II plus p24 I/II or plus a gp46 I/II pattern. In addition, 226 samples with available blood cells were tested by qPCR; these samples included 29 HTLV-1 positive samples, 5 HTLV positive samples, 59 indeterminate samples, and 133 negative samples. It was discovered that all of the LIA negative samples could not be detected by qPCR, but 93.1% (27/29) HTLV-1 samples, 40% (2/5) HTLV-untyped samples, and 1.7% (1/59) indeterminate samples could all be detected. Consequently, 50 donors were determined to be HTLV-1/2 carriers (Table 1). The average prevalence in areas except for Fujian, Zhejiang, and most cities in Guangdong was only 1.123/100,000 (50/4,451,883, 95% CI 2.108–2.805). Additionally, all samples that could be detected using qPCR were amplified for the LTR region using nested PCR, and 89.7% (26/30) were successfully amplified and sequenced. The final results and validation algorithms are displayed in Figure 1 and Table 1.

**Table 1:** The clinical features and results of LIA, qPCR, and nested PCR among the finally confirmed HTLV-1/2 positive individuals.

**Number**	**Province (or City)**	**Sex**	**Age**	**INNO-LIA**	**qPCR**		**LTR**
				**Result**	**Copies/100 Cells**	**Result**	**Phylogenetic Analysis**
**1918**	Shenzhen	54	Male	HTLV-1	5.288	HTLV-1	Transcontinental
**1922**	Shenzhen	52	Female	HTLV-1	1.110	HTLV-1	Japanese
**2722**	Hunan	50	Male	HTLV-1	6.886	HTLV-1	Japanese
**2751**	Hunan	35	Male	HTLV-1	4.002	HTLV-1	Japanese
**2415**	Hunan	52	Male	HTLV	0.533	HTLV-1	Japanese
**1705**	Jiangxi	40	Female	HTLV-1	11.400	HTLV-1	Japanese
**2541**	Jilin	44	Male	HTLV-1	1.114	HTLV-1	Japanese
**2550**	Hainan	60	Male	HTLV-1	1.956	HTLV-1	Transcontinental
**2649**	Gansu	58	Female	HTLV-1	0.010	HTLV-1	Transcontinental
**2701**	Jiangsu	28	Female	HTLV-1	0.132	HTLV-1	Transcontinental
1626	Tianjin	42	Male	HTLV-1	0.339	HTLV-1	Transcontinental
1757	Jilin	57	Female	HTLV-1	2.278	HTLV-1	Transcontinental
1785	Jiangxi	43	Female	HTLV-1	3.001	HTLV-1	Japanese
2250	Shenzhen	22	Female	HTLV-1	0.670	HTLV-1	Transcontinental
2251	Shenzhen	43	Female	HTLV-1	0.795	HTLV-1	Japanese
2271	Shenzhen	33	Female	HTLV-1	0.120	HTLV-1	Transcontinental
2362	Jilin	53	Female	HTLV-1	0.026	HTLV-1	Japanese
2368	Jiangxi	23	Female	HTLV-1	0.071	HTLV-1	Transcontinental
2394	Hebei	49	Male	HTLV-1	0.678	HTLV-1	Japanese
2406	Guangxi	31	Female	HTLV-1	1.230	HTLV-1	Transcontinental
2548	Hainan	64	Male	HTLV-1	0.033	HTLV-1	Japanese
2606	Jiangxi	55	Male	HTLV-1	0.913	HTLV-1	Transcontinental
2613	Shandong	32	Female	HTLV-1	2.815	HTLV-1	Transcontinental
2718	Shenzhen	41	Male	HTLV-1	0.695	HTLV-1	Japanese
2752	Shanghai	41	Female	HTLV-1	1.562	HTLV-1	Transcontinental
2840	Jiangxi	33	Male	HTLV-1	2.254	HTLV-1	Japanese
**1782**	Shandong	25	Male	HTLV-1	0.273	HTLV-1	nPCR negative
1739	Jiangxi	53	Female	HTLV	0.009	HTLV-1	nPCR negative
2412	Guangxi	45	Female	HTLV-1	0.001	HTLV-1	nPCR negative
2519	Hubei	42	Male	IND	0.017	HTLV-1	nPCR negative
2467	Guizhou	37	Male	HTLV-1	ND	Negative	nPCR negative
2719	Hebei	38	Female	HTLV-1	ND	Negative	nPCR negative
1747	Anhui	23	Male	HTLV	ND	Negative	nPCR negative
1951	Hebei	22	Male	HTLV	ND	Negative	nPCR negative
2657	Hebei	37	Male	HTLV	ND	Negative	nPCR negative
**1598**	Shenzhen	34	Female	HTLV-1	NA		NA
1594	Yunnan	53	Male	HTLV-1	NA		NA
1977	Hebei	35	Male	HTLV-1	NA		NA
1994	Heilongjiang	48	Female	HTLV-1	NA		NA
2131	Sichuan	51	Male	HTLV-1	NA		NA
2373	Guangxi	41	Male	HTLV-1	NA		NA
2446	Heilongjiang	57	Male	HTLV-1	NA		NA
2634	Shenzhen	19	Male	HTLV-1	NA		NA
2755	Shandong	22	Female	HTLV-1	NA		NA
1965	Yunnan	47	Female	HTLV	NA		NA
2363	Jilin	50	Male	HTLV	NA		NA
1607	Guizhou	NA		HTLV-1	NA		NA
1829	Guizhou	NA		HTLV-1	NA		NA
2629	Shenzhen	NA		HTLV-1	NA		NA
2280	Guizhou	NA		HTLV-1	NA		NA

LIA, line immunoassay; IND, indeterminate; ND, not detectable; nPCR, nest PCR; NA, not available. Bold numbers indicate that the infected person answered the questionnaires.

### Characteristics of the HTLV-1-infected individuals

Among the 50 confirmed HTLV1/2-infected individuals, 46 provided demographic information (age, sex, donation history, ethnicity, and blood type). Supplementary Table S2 summarizes the most relevant clinical and demographic features of these individuals. The mean age was 41.61 years, and 67.4% ranged from 36 to 55 years. Besides, 54.3% were men, and 45.7% were repeat donors. A total of 12 HTLV-1-infected patients agreed to answer the questions listed on the questionnaire (Supplementary Table S3). We found that one in three cases from Shenzhen had lived in Fujian Province for a long time, and 5 out of 9 individuals had lived in Fujian, Guangdong, or Zhejiang for more than 3 months. Moreover, Fujian Province is the birthplace of two of the 34 infected individuals (without questionnaire demographic information). Regarding personal experiences of possible HTLV-1/2 infection, 75% (9/12) of the surveyed individuals had experienced extraction, filling, or fixing of teeth; 8.3% (1/12) had received a blood transfusion; and 58.3% (7/12) had received surgical or suture treatment. Of these, only 16.7% (2/12) did not have any of the aforementioned experiences. Of 12 infected patients, 4 had 3–4 sexual partners, 6 had only 1 sexual partner, and 2 refused to provide this information. Regarding HTLV-1-related symptoms or diseases, one of the surveyed persons was diagnosed with infectious dermatitis and bronchial lung disease, one was diagnosed with chronic inflammatory arthritis, and one had skin itching symptoms. Therefore, 75.0% (9/12) of infected patients were asymptomatic carriers.

### HTLV-1 subtypes and distributions

**Figure 2:**
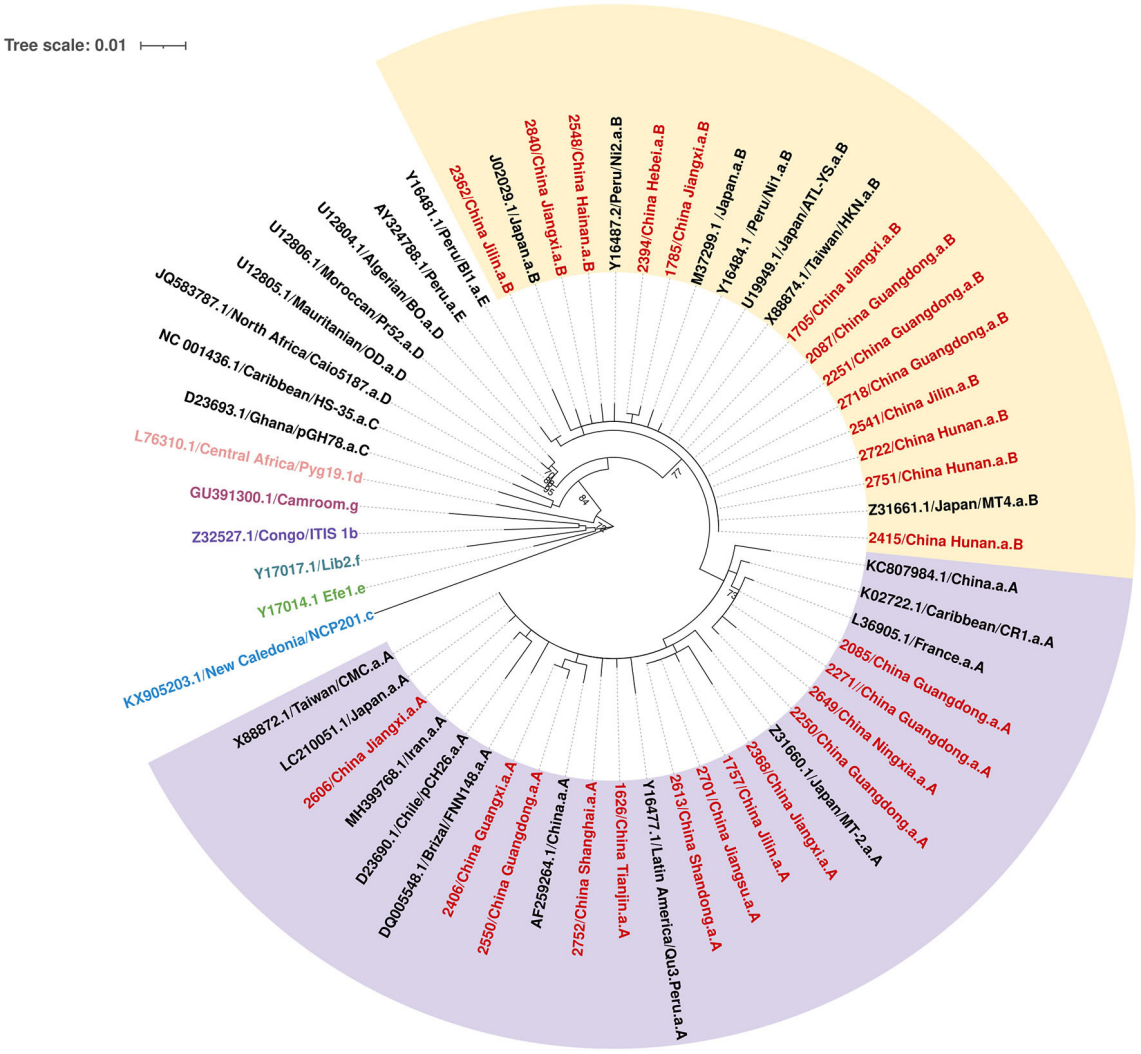
Phylogenetic tree based on the 700-bp nucleotide sequence of the LTR region of the HTLV-1, using the maximum likelihood approach, with 1,000 bootstrap replications, repeated 10 times.

**Figure 3:**
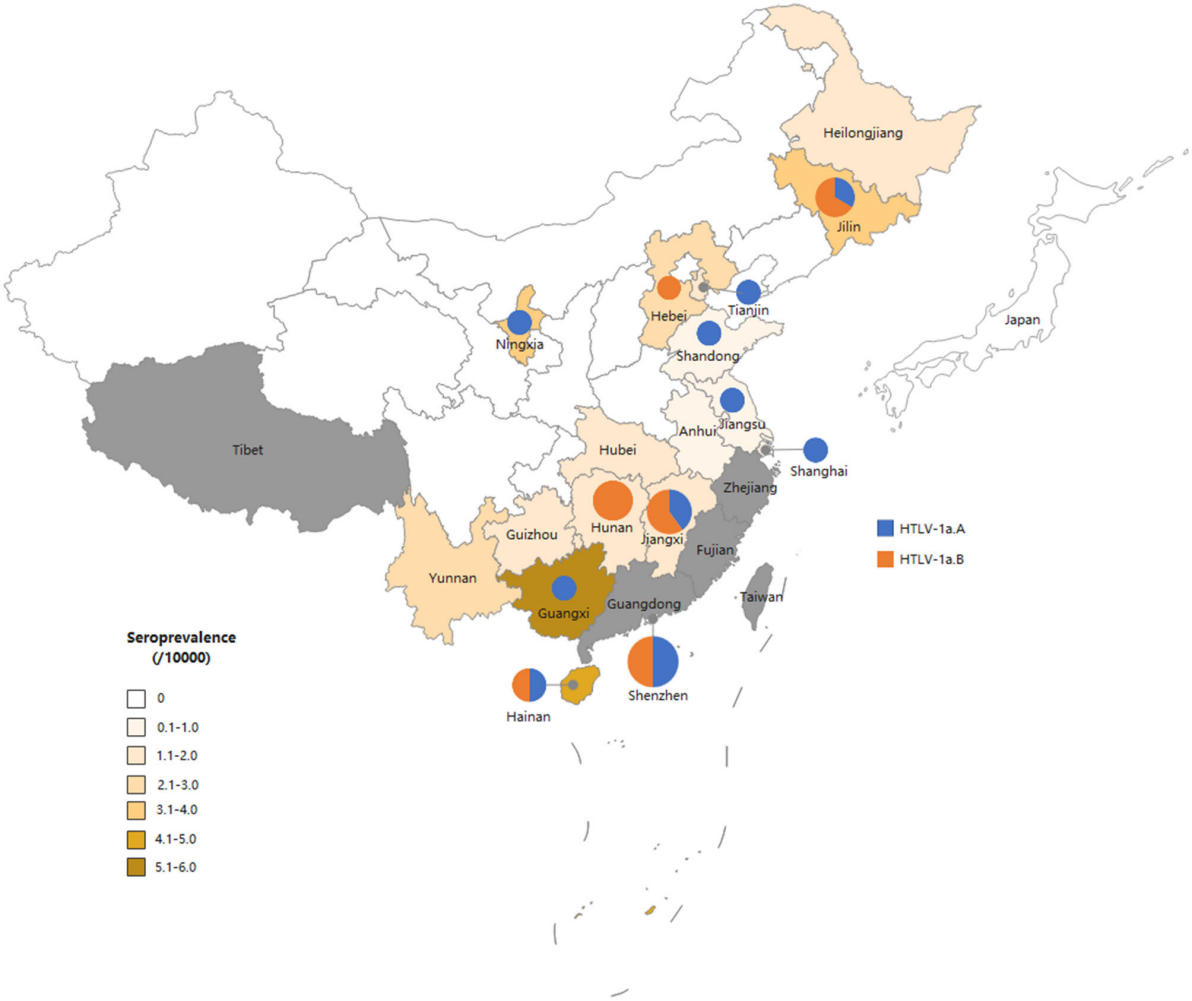
Map of China showing the general distribution of HTLV-1 genotypes across the country. The size of the circles is proportional to the number of strains identified in this study. The smallest size corresponds to one characterized strain and the largest to six strains.

A total of 50 individuals finally confirmed HTLV-1/2 infection in 18 provinces, of which 26 samples collected from 12 provinces were successfully sequenced. As displayed in Figure 2, all HTLV-1 isolates belonged to the Cosmopolitan (1a) subtype. Among them, 50% (13/26) of HTLV-1 isolates belonged to the Transcontinental (A) subgroup, and the others belonged to the Japanese (B) subgroup. Interestingly, the Transcontinental subgroup was distributed in 83.3% (10/12) of the provinces, and seven occurred only in subgroup A; however, only one isolate has been analyzed in each of these provinces (Figure 3 and Supplementary Table S4). The Japanese subgroup was found in 50% (6/12) of the provinces, and the proportion was ≥50% in each province, including Jilin, Hebei, Hunan, Jiangxi, Guangdong, and Hainan provinces. Remarkably, three individuals from Hunan Province belonged to the Japanese subgroup, but they all lived in Fujian, Zhejiang, or Guangzhou provinces for at least 3 months.

### HTLV-1 infection among relatives

**Figure 4:**
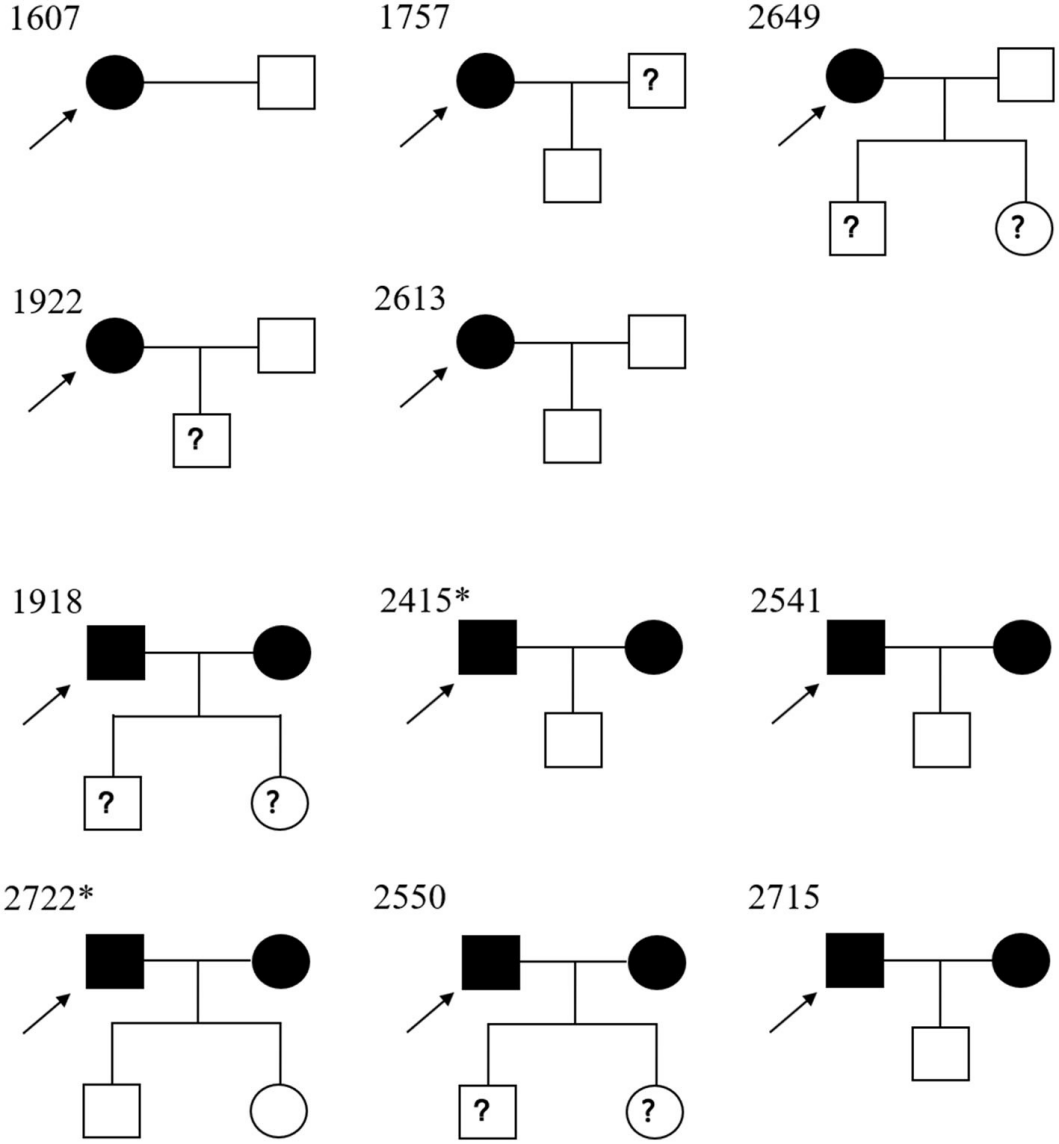
Pedigree of the 11 families of Chinese descendants under study. Square, male; circle, female. Black full square/circle, HTLV-1-infected individual; empty full square/circle, uninfected individual; ? square/circle, unknown HTLV-1 status. → , index case. *Their children were breastfed for more than 10 months.

All families of HTLV-1/2 positive individuals were contacted, and 11 agreed to allow their relatives to be tested. In this study, 17 samples were collected from family members of those HTLV-1-infected blood donors. Five index cases were women, and the others were men. As illustrated in Figure 4 and Table 2, the prevalence of HTLV-1 in wives (83.3%, 5/6) was much higher than in husbands (0.0%, 0/4); the difference was significant (*p* < 0.05). Seven of the index cases provided information about how many sexual partners they had had. Two of them (index 2649 and 2751) had previously had 3–4 sexual partners, and the others (index 1922, 1918, 2541, 2722, and 2550) had only one. As for the vertical transmission, seven kids' blood samples were tested, but none were positive for HTLV-1/2 antibodies. Interestingly, four children who had been breastfed for more than 10 months by their infected mothers were not infected. Additionally, index case 2,613 was obtained from the Shandong Blood Hematopoietic Stem Cell Bank, and her child's umbilical cord blood was LIA-HTLV-1 positive but could not be detected using qPCR. While her baby's blood sample was serologically negative.

**Table 2:** Frequency of HTLV-1 infection in mother-offspring and spouse-spouse pairs in this study.

**Route of transmission**	**Number of subjects studied**	**Numbers of subjects infected with HTLV-1(%)**	***p*-value**
**Vertical**			
Daughters or sons	7	0 (0.0)	/
**Sexual**			
Wives	6	5 (83.3)	0.01
Husbands	4	0 (0.0)	

## Discussion

The devastating diseases induced by HTLV-1 have a greater impact on health than is commonly cited, indicating that virus infection should not be neglected. In China, in the early 1980s, HTLV-1/2-infected cases were found in three Japanese and two Taiwanese patients, in one ATL patient, and in two Chinese women whose husbands were positive Japanese and Taiwanese individuals (Zeng et al., 1984). Later, during the 1990s and early 2000s, HTLV-1/2 infection was restricted to Fujian Province (Geng et al., 1998; Wang et al., 2005). From the late 2000s to 2015, HTLV-1/2 infection was first detected in other provinces, including Guangdong, Zhejiang, Henan, and Hubei (Ma et al., 2013). A recent study screened anti-HTLV antibodies among blood donors from 2016 to 2018, which indicated a larger expansion of HTLV-1/2 infections in China (Chang et al., 2021). According to that report, most areas in China, except Fujian and Zhejiang provinces, belong to non-endemic areas with an average prevalence of 1.736/100,000, which was slightly higher than that displayed in our study. This may be due to the permanent ban on blood donation from HTLV-1/2 positive blood donors in recent years. In our study, areas geographically adjacent to Fujian, such as Hunan, Jiangxi, and Guangxi provinces, still demonstrated relatively high HTLV-1/2 prevalence (Chang et al., 2021). In addition, some regions that never reported HTLV-1/2-infected individuals existed in sporadic cases, including Hainan, Anhui, Ningxia, and Shandong provinces, suggesting a silent and slow spread of HTLV-1/2 throughout the country. Therefore, screening for HTLV-1/2 antibodies should be performed routinely in regions with a relatively high prevalence of HTLV-1/2 to prevent its transmission.

A total of 50 individuals were finally confirmed to be HTLV-1/2 positive in this study. The average age of the positives was 41.6 years, and 71.7% of them were over 35 years, which was similar to 69.5% reported in Guangdong Province (Liao et al., 2021) but was much higher than 30.0% reported in Fujian Province. However, according to China's report on blood safety in 2019, the proportion of blood donors over 35 years old was ~47.5% in Fujian Province and ~52.7% of blood donors nationwide. These data indicate that HTLV-1/2 infection occurs in young people in Fujian Province but mainly in elderly people in other areas. This phenomenon is difficult to explain, but it may be related to the early introduction of HTLV-1/2 into Fujian, where the younger generation has been transmitted through various routes for a long time. The raised prevalence among older people could be explained by the high rate of sexual transmission, which is consistent with the high rate of likely male-to-female transmission observed in this study. As for other regions, a new generation of infected individuals has not emerged. If there is no intervention, the age of infected people is likely to decline over many years. In this study, 45.7% of infected subjects were women, but the prevalence in women was higher than that in men because ~37% of blood donors were women. Our data support the finding that female sex is a strong risk factor for HTLV-1 positivity (Satake et al., 2016; Jensen et al., 2019).

All HTLV-1/2 carriers were contacted to complete a questionnaire, and we received 12 responses. We found that not all infected blood donors were asymptomatic carriers since three had HTLV-1-related symptoms, such as arthritis, infective dermatitis, and bronchopneumonia. Although HTLV-1 can cause local and systemic inflammatory diseases, a direct correlation between these symptoms and HTLV-1 infection requires further investigation. HTLV-1 is believed to occur through contact from mother to child, predominantly *via* breastfeeding, and through exposure to contaminated blood. We found that most of the investigated blood donors underwent invasive procedures, such as surgery, transfusion, acupuncture, or teeth washing, and one of them received a blood transfusion. These experiences have increased the possibility of virus infection. Interestingly, most HTLV-1/2 carriers have lived in Fujian, Zhejiang, or Guangdong provinces for over 3 months, indicating that they might have been infected in these areas.

Our study genotyped HTLV-1 isolates from non-endemic areas in China for the first time. Molecular analyses demonstrated that all blood donors presented the HTLV-1a genotype, of which half belonged to the Transcontinental subgroup and the rest clustered within the Japanese subgroup. The proportion of Transcontinental subgroups in our study was lower than that reported previously (Wang et al., 2005; Xie et al., 2015; Liao et al., 2021). Before 2010, all isolates of HTLV-1 from Fujian Province belonged to the Transcontinental subgroup (Wang et al., 2005). In 2015, Xie et al. (2015) first reported two Japanese subtypes among 27 HTLV-1 carriers enrolled in Fujian Province. In 2021, Liao et al. (2021) demonstrated that 16 isolates (16/55, 29.1%) from Guangdong belonged to the Japanese subtype. The Transcontinental subgroup was supposed to have migrated to China before the Japanese subgroup (Liao et al., 2021). Previous studies have indicated that some HTLV-1 forms belonging to the Transcontinental subgroup are related to the Asian mongoloid HTLV-1 (Sonoda et al., 2011). This subtype may have been transmitted for a long time in Fujian and then gradually spread to neighboring cities. In fact, Fujian was the birthplace of three HTLV-1-infected cases in our study, two of which were sequenced, and all belonged to the Transcontinental subgroup, consistent with previous reports. As for the Japanese subgroup, it was unclear about the earliest transmission of this subgroup, but geographically, Japan is adjacent to the southeastern coast of Guangdong and Fujian provinces, as well as the northeastern provinces in China such as Jilin and Heilongjiang provinces. Two of three HTLV-1-infected patients in Jilin Province belonged to the Japanese subgroup, which agreed with our speculation. Guangdong Province is a prosperous metropolis where large-scale population movement increases the risk of imported HTLV-1/2 infection and induces its transmission to other low-prevalence areas. A previous study indicated that all infected people in Shenzhen were imported from Fujian and the adjacent eastern Guangdong regions (Liao et al., 2021). Therefore, inland provinces adjacent to Guangdong exhibited relatively higher prevalence rates than other provinces and were also introduced to the Japanese subtype. Our results generally indicated that the predominant HTLV-1 subgroup in China was the Transcontinental subgroup, but the Japanese subgroup was distributed geographically. However, further phylogenetic evaluations with larger sample sizes are required.

To the best of our knowledge, this is the first study to investigate the familiar transmission of HTLV-1 infection in the non-endemic areas of China. We evaluated 11 HTLV-1-infected cases and 17 family members. As presented in Figure 4 and Table 2, the seroprevalence in wives with seropositive husbands (83.3%) was greater than that in husbands with seropositive wives (0.0%), which suggested the possible sexual transmission of HTLV-1 (predominantly male-to-female transmission). These data agree with previous data observed in Japan (Tajima et al., 1982; Stuver et al., 1993), and support the fact that HTLV-1 infection rates are higher in women than in men (da Costa et al., 2013; Nozuma et al., 2014; Frutos et al., 2017). HTLV-1 is also transmitted *via* vertical (breastfeeding) routes. Prospective studies have shown an average vertical transmission rate of 20.5% among children breastfed for ≥6 months, 8.3% among those breastfed for <6 months, and 2.4% among those fed by other routes (Percher et al., 2016). However, 4 of the 7 analyzed children had been breastfed over 10 months, and no children had been vertically infected in this study. A possible explanation is that these HTLV-1-carrier mothers were infected after they breastfed their children. Bandeira et al. (2018) reported similar results among Japanese immigrants in Brazil, suggesting that current HTLV-1 transmission is less effective than before but deserves further investigation (Bandeira et al., 2018). Additionally, index case 2613 was a young woman who donated cord blood to the Shandong Blood Hematopoietic Stem Cell Bank. The plasma from the cord blood displayed a strong reaction with the specific bands but a qPCR-undented result. This may largely be due to the defective or non-infectious HTLV-1 proviruses in the cord blood (Katamine et al., 1994; Kazi et al., 1998; Glasser et al., 2013).

In summary, although HTLV-1/2 prevalence in non-endemic regions in China is as low as previously reported, an effective HTLV-1/2 screening strategy should be adopted to reduce the risk of transmission from the southern coastal areas of China as well as other highly endemic countries. From the molecular analysis, the Transcontinental subgroup may be the predominant HTLV-1 subgroup in the southeastern coastal areas of China, but not in the northeastern coastal areas or other regions. With the expansion of international trade and the increased population migration rate, the Japanese subtype may gradually become more prevalent in some regions. Moreover, a higher transmission risk was observed from male to female than *vice versa*, and younger generations were less susceptible to infection. However, the general population is vulnerable to being infected without a prophylactic vaccine and effective treatment of HTLV-1/2-related diseases. Therefore, in addition to regular HTLV-1/2 antibody testing, counseling of the virus carriers is critical in preventing further HTLV-1/2 transmission.

## Data availability statement

The original contributions presented in the study are included in the article/Supplementary material, further inquiries can be directed to the corresponding author.

## Ethics statement

The studies involving humans were approved by the Beijing Hospital Ethics Committee. The studies were conducted in accordance with the local legislation and institutional requirements. Written informed consent for participation in this study was provided by the participants' legal guardians/next of kin.

## Author contributions

HJ: Data curation, Investigation, Writing—original draft. LC: Investigation, Methodology, Writing—review and editing. YY: Methodology, Validation, Writing—review and editing. HS: Investigation, Methodology, Writing—original draft. YL: Software, Validation, Writing—review and editing. LW: Validation, Writing—review and editing.
